# The role of lipid-modified proteins in cell wall synthesis and signaling

**DOI:** 10.1093/plphys/kiad491

**Published:** 2023-09-08

**Authors:** Oliver Quinn, Manoj Kumar, Simon Turner

**Affiliations:** Faculty of Biology, Medicine and Health, University of Manchester, Michael Smith Building, Dover Street, Manchester M13 9PT, UK; Faculty of Biology, Medicine and Health, University of Manchester, Michael Smith Building, Dover Street, Manchester M13 9PT, UK; Faculty of Biology, Medicine and Health, University of Manchester, Michael Smith Building, Dover Street, Manchester M13 9PT, UK

## Abstract

The plant cell wall is a complex and dynamic extracellular matrix. Plant primary cell walls are the first line of defense against pathogens and regulate cell expansion. Specialized cells deposit a secondary cell wall that provides support and permits water transport. The composition and organization of the cell wall varies between cell types and species, contributing to the extensibility, stiffness, and hydrophobicity required for its proper function. Recently, many of the proteins involved in the biosynthesis, maintenance, and remodeling of the cell wall have been identified as being post-translationally modified with lipids. These modifications exhibit diverse structures and attach to proteins at different sites, which defines the specific role played by each lipid modification. The introduction of relatively hydrophobic lipid moieties promotes the interaction of proteins with membranes and can act as sorting signals, allowing targeted delivery to the plasma membrane regions and secretion into the apoplast. Disruption of lipid modification results in aberrant deposition of cell wall components and defective cell wall remodeling in response to stresses, demonstrating the essential nature of these modifications. Although much is known about which proteins bear lipid modifications, many questions remain regarding the contribution of lipid-driven membrane domain localization and lipid heterogeneity to protein function in cell wall metabolism. In this update, we highlight the contribution of lipid modifications to proteins involved in the formation and maintenance of plant cell walls, with a focus on the addition of glycosylphosphatidylinositol anchors, N-myristoylation, prenylation, and S-acylation.

## Introduction

The plant cell wall is a polysaccharide-rich extracellular matrix that surrounds cells. The composition and organization of the wall are highly dynamic, differing during development and between species and cell types (Gigli-Bisceglia et al. 2020). The primary cell wall is a relatively thin and flexible layer that supports cell expansion while resisting turgor pressure and protecting the cell against bacterial and fungal infection (Cosgrove 2005). In contrast, only specialized cells have a secondary cell wall, which provides the plant with structural support and allows water transport in xylem tissues (Bellincampi et al. 2014). Cell walls are also modified in response to biotic and abiotic stresses; these changes include the reinforcement of the wall to improve its performance as a barrier to microbes and changes that conserve energy and resources when light and nutrient conditions are limiting (Houston et al. 2016; Wang et al. 2016). Beyond being a protective layer, the cell wall is also the site of intracellular signaling, imposes a limitation on the diffusion of proteins at the plasma membrane, and contributes to the architecture of the cell (Zhong et al. 2019; Gigli-Bisceglia et al. 2020).

**Figure 1.**
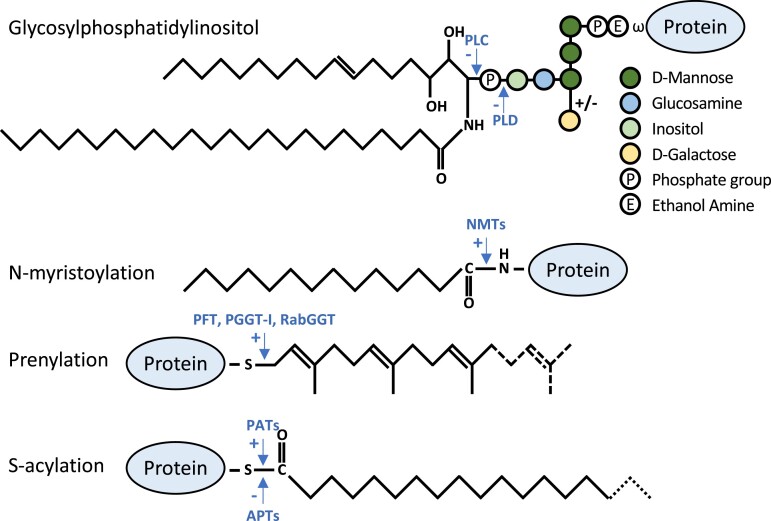
Structures and important features of lipid modifications. Glycosylphosphatidylinositol anchor modification is the addition of a glycolipid by amide linkage, at the “ω” site, following the cleavage of a C-terminal hydrophobic signal sequence. Various lipid and glycan structures exist; the glycosylphosphatidylinositol anchor shown is based on the structure determined in Oxley and Bacic (1999). Cleavage sites for phospholipase C (PLC) and D (PLD) are marked by arrows. N-myristoylation is the irreversible addition of a 14-carbon myristate acyl lipid group, by NMTs, through an amide linkage to a glycine residue at position 2 following the cleavage of the initiator methionine. Prenylation is the irreversible addition of 15- carbon farnesyl (solid lines) or 20-carbon geranylgeranyl (dashed lines) isoprene lipid moieties through thioether linkage to C-terminal cysteines at various motifs, including CaaX, where “a” are aliphatic amino acids and “X” can be any amino acid. Alternatively, prenylation can occur in the absence of a CaaX motif by the interaction of the modified protein with REP. Multiple enzymes catalyze the addition of prenyl lipids; PFT, PGGT-I, and RabGGT. S-acylation is the addition of 16-carbon palmitate (solid lines) or 18-carbon stearate (dashed lines) acyl lipid to cysteine residues through a thioester linkage by PATs. S-acylation is not limited to protein N or C termini; there is little, or no, obvious consensus sequence around the modified cysteines. Unlike other lipid modifications, S-acylation is reversible through the action of APTs.

In angiosperms, primary cell walls are composed largely of cellulose, xyloglucan, and pectin, while the major components of secondary cell walls are cellulose, xylan, and lignin (Gigli-Bisceglia et al. 2020). Both types of cell wall also contain cell wall proteins. Despite making up less than 10% of the cell wall by mass, proteins are essential in cell wall structure and biogenesis. The roles of cell wall proteins include modifying polysaccharides, binding polysaccharides, and interacting with plasma membrane-localized receptors. These include proteins such as expansins (Cosgrove 2016; Narváez-Barragán et al. 2022), arabinogalactan proteins (AGPs) (He et al. 2019; Lin et al. 2022a, b, c) and pectin methylesterases (PMEs) (Shin et al. 2021). Many proteins localized within the cell wall and involved in the synthesis and delivery of cell wall components to the apoplastic space are modified with lipid groups. These lipid modifications include the addition of a glycosylphosphatidylinositol (GPI) anchor, N-myristoylation, prenylation, and S-acylation (Fig. 1). The function of each of these lipid modifications varies at least partly due to their targeting of proteins to different sides of the plasma membrane. GPI anchors tether proteins to the outer leaflet of the plasma membrane, whereas the other lipid modifications interact with the inner leaflet of the plasma membrane and membranous organelles (Giglione and Meinnel 2022; Xu et al. 2022a, 2022b). Lipid modification of many of these proteins is essential for their function in cell trafficking (Kumar et al. 2016; Rojek et al. 2021), membrane anchorage or interaction (Basu et al. 2016; Feng et al. 2018), protein-protein interaction (Mahajan et al. 2008), and membrane microdomain localization by promoting the clustering of similarly modified proteins to membrane regions with distinct lipid compositions (Xu et al. 2022a, 2022b; Stroppa et al. 2023). Evidence for the importance of lipid modification of proteins for plant cell wall synthesis and/or structure comes from studies that have disrupted the enzymes involved in lipid metabolism, which could be the underlying cause of resulting cell wall defects (Hemsley et al. 2005; Renna et al. 2013; Zhang et al. 2015; Bernat-Silvestre et al. 2021; Gutkowska et al. 2021).

Many proteins also bear multiple lipid modifications, being either dual lipid-modified or modified with the same modification at multiple sites. Dual lipid-modified proteins often rely on an irreversible modification such as prenylation or N-myristoylation to promote interactions with membranes, and a second modification such as S-acylation is required for localization and function (Li et al. 2022; Park et al. 2023). In this article, we summarize the roles of lipid modification of proteins in the regulation of cell wall deposition and its response to the environment.

## GPI-anchored proteins

The attachment of proteins to the outer leaflet of the plasma membrane through C-terminal modification with a GPI anchor is conserved in eukaryotes. In plants, this results in protein localization to the apoplastic space. Recent genetic and proteomic studies predict that 248 Arabidopsis (*Arabidopsis thaliana*) proteins bear a GPI modification (Rui and Dinneny 2020). Following the attachment of the preassembled GPI anchor to a protein, the anchor undergoes further lipid and glycan remodeling, understood to be important for the processing of GPI-anchored proteins through the secretory system toward their final localization (Bernat-Silvestre et al. 2021). For some GPI-anchored proteins, the lipid modification can be cleaved, releasing the protein from the outer leaflet of the plasma membrane and allowing it to move into the apoplast (Sedbrook et al. 2002; Xue et al. 2017). Remodeling of the GPI anchor in the Golgi promotes clustering of similarly anchored proteins and aids their sorting during secretion to the apoplastic space (Xu et al. 2022a, 2022b). Currently, only 1 plant GPI anchor structure has been determined (Oxley and Bacic 1999). This structure, obtained from *Pyrus communis* suspension-cultured cells, confirmed that the core glycan is conserved between eukaryotes. Following the addition of a GPI anchor to a protein in the endoplasmic reticulum (ER), the GPI lipid moiety undergoes extensive remodeling; the steps of this are well established in yeast and mammals (Kinoshita et al. 2008; Kinoshita 2020). Many mammalian and yeast GPI lipid-modifying enzymes have orthologs in plants, and through studying these genes progress has been made in understanding GPI lipid remodeling in plants. In the Arabidopsis inositol deacylase mutant *POST-GPI ATTACHMENT TO PROTEINS 1* (*PGAP1*), the exit of GPI-anchored proteins from the ER and delivery to the plasma membrane is delayed (Bernat-Silvestre et al. 2021). Interestingly, removal of the acyl group from the inositol ring by PGAP1 was shown to be required for the effective release of GFP-tagged SKU5 by cleavage of the GPI anchor of SKU5, suggesting that the lipid moiety of the GPI anchor is important for phospholipase recognition (Lin et al. 2022a, 2022b, 2022c). Another GPI lipid remodeling enzyme, a membrane-bound O-acyltransferase, was identified as rice (*Oryza sativa*) Brittle Culm 16 (OsBC16) (Xu et al. 2022a, 2022b). OsBC16 was found to be essential for targeting GPI-anchored OsBC1 to lipid nanodomains, where it functions in cellulose synthesis; this is likely due to the acyltransferase activity of OsBC16 during GPI lipid remodeling (Xu et al. 2022a, 2022b).

**Figure 2.**
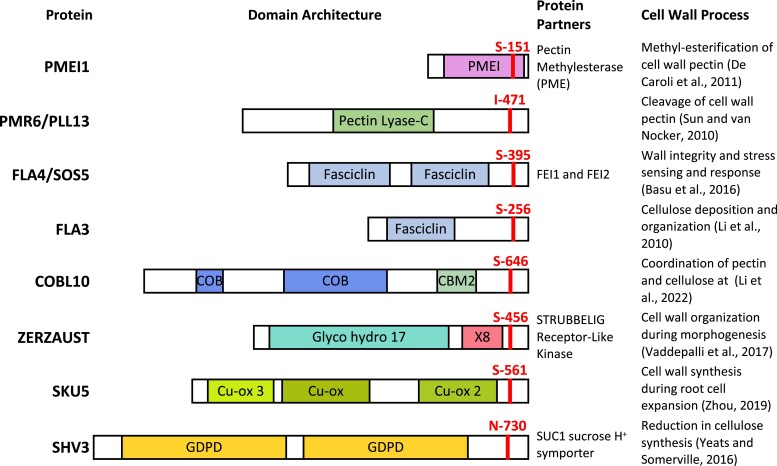
Domain organization of a selection of GPI-anchored proteins involved in cell wall regulation. A diagrammatic representation of the domain architecture of several GPI-anchored proteins involved in cell wall–related processes. Domain organization is based on protein sequences searched using CDvist (Adebali et al. 2015). CBM2, carbohydrate-binding type-2; GDPD, glycerophosphodiester phosphodiesterase; Glyco hydro 17, Glycoside hydrolase family 17; The ω sites predicted using PredGPI are marked in red (Pierleoni et al. 2008).

The essential role of GPI-anchored proteins in the regulation of cell wall structure and composition was demonstrated by *peanut1* (*pnt1*) mutants caused by a defect in a homologue of PHOSPHATIDYLINOSITOL GLYCAN OF COMPLEMENTATION CLASS M (PIG-M). PIG-M is an endoplasmic reticulum–localized mannosyltransferase required for the synthesis of GPI anchors. *pnt1* mutants exhibit reduced crystalline cellulose content, increased pectin deposition, and ectopic cell wall deposition and are seedling lethal (Gillmor et al. 2005). Understanding the role of GPI-anchored proteins in the cell wall is complicated by the large number of proteins modified in this way. While many GPI-anchored proteins appear essential for normal cell wall metabolism and signaling, many share only the anchor in common and represent a diverse set of protein structures (Fig. 2). Detailed reviews of GPI-anchored proteins are available (Desnoyer and Palanivelu 2020; Silva et al. 2020; Zhou 2022). Here we focus on selected examples that illustrate recurring themes regarding the role of GPI-anchored proteins in cell wall metabolism and signaling.

### Arabinogalactan proteins (AGPs) and Fasciclin-like arabinogalactan proteins

AGPs comprise a large family of highly glycosylated, hydroxyproline-rich proteins that bear GPI anchors. They are localized in the apoplast, where they contribute to cell wall structure and function as signaling intermediates (Xue et al. 2017; Seifert 2018). One of the best-studied examples is the classical AGP, Arabinoxylan Pectin Arabinogalactan Protein 1 that integrates into the cell wall through covalent linkage of their arabinogalactan domains to cell wall hemicellulose and pectin (Tan et al. 2013). A subgroup of the AGPs also contains a Fasciclin-like (FAS) domain (Fig. 2). FAS domains are found in all kingdoms and act as cell adhesion domain in mammals, giving rise to the idea that Fasciclin-like AGPs (FLAs) function as putative sensors of cell wall integrity (MacMillan et al. 2010; He et al. 2019). Both expression and mutant data suggest FLAs are likely to possess additional roles in cell wall synthesis (see below). There are 21 FLAs in Arabidopsis and up to 50 in poplar, many of these arose due to gene duplication events and therefore redundancy likely masks FLA mutant phenotypes (Showalter et al. 2016); however, phenotypes associated with mutation in several FLA genes have been reported.

FLA4 was initially identified as Salt Overly Sensitive 5 during a screen for mutants with increased salt sensitivity and recognized as an AGP. FLA4 acts in a linear genetic pathway with a pair of functionally redundant leucine-rich repeats receptor-like kinases: FEI1 and FEI2 (Seifert et al. 2014; Basu et al. 2016). The salt-hypersensitive *fla4* mutants have thinner cell walls, swollen root cells, and reduced root length following salt treatment (Shi et al. 2003). This phenotype is mirrored in the *fei1/2* double mutant and is non-additive, with *fla4,* suggesting that they work in the same pathway. Growth of *fla4* mutants is comparable to wild type under normal growth conditions, further suggesting that FLA4 acts during the response to salt stress; this is likely mediated by the predicted interaction of the C-terminal FAS domain of FLA4 with the leucine-rich receptor kinase FEI1 (Turupcu et al. 2018). FLA4 lacking the GPI anchor is largely retained in the ER. The *fla4* mutant is complemented by FLA4 lacking the GPI anchor; this is likely because sufficient FLA4 is still able to reach the cell surface or is secreted. FLA4 likely acts predominantly within the cell wall, and its anchorage in the membrane probably promotes clustering with receptors or allows its controlled release into the wall by cleavage under specific circumstances, the details of which are not understood (Xue et al. 2017). Similarly, when the GPI modification is disrupted by a mutation in PGAP1, FLA11 is retained in the ER and Golgi (Bernat-Silvestre et al. 2021). The cell wall of the *pgap1-3* mutant also has increased arabinan and reduced type II arabinogalactan and xyloglucan, emphasizing the potential role of GPI modification in both normal FLA11 targeting and cell wall construction (Bernat-Silvestre et al. 2021).

Overexpression of FLA11 resulted in premature secondary cell wall deposition, with the resultant cell wall having increased lignification and reduced crystalline cellulose content, similar to the typical changes in the cell wall following compression stress. This implicates FLA11 as a sensor of cell wall integrity, although it remains unclear how the absence of functional FLA11 results in reduced stem tensile strength (MacMillan et al. 2010; Ma et al. 2022). This large change in mechanical properties of *fla* mutants suggests a more fundamental role for FLA11/12 in determining cell wall structure (MacMillan et al. 2010).

Several other FLAs are GPI anchored and involved in the regulation of the cell wall during development (Johnson et al. 2003; Liu et al. 2020). FLA3 disruption by RNA interference results in aberrant cellulose deposition in the pollen intine cell wall as well as shrunken and wrinkled pollen grains (Li et al. 2010). Similar pollen tube growth phenotypes are seen in mutants of 2 subunits of the N-acetylglucosaminyltransferase required for the synthesis of the GPI anchor, suggesting that the GPI anchor of FLA3 is important for its function, potentially by promoting the clustering of GPI-anchored receptors at the plasma membrane (Lalanne et al. 2004).

### Pectin modification by GPI-anchored enzymes

Pectins are a diverse group of polysaccharides with a common core of 1,4-linked α-D-galacturonic acid found predominantly in the primary cell wall, where they contribute to the formation of a gel-like matrix surrounding the cellulose fibrils (Voragen et al. 2009; Shin et al. 2021). Synthesized in the Golgi, pectins are secreted into the apoplastic space, where they are further modified, altering the porosity and surface charge of the wall (Shin et al. 2021). Newly synthesized homogalacturonan domains of pectin can be demethylated by pectin methylesterases (PMEs), resulting in increased cell wall flexibility that has been shown at the shoot apical meristem and is likely to be a feature of many cell types (Braybrook and Peaucelle 2013). Numerous PMEs and PME inhibitors (PMEIs) are predicted to bear GPI anchors. Currently, only PMEI1 has been experimentally confirmed to be GPI anchored, with GPI modification potentially facilitating its Golgi exit and secretion to the apoplast. The requirement for the GPI anchor of PME1 has not yet been experimentally validated (De Caroli et al. 2011). To date, the most likely potential function of GPI anchoring of PMEs and PMEIs is for their sorting and secretion to the outer leaflet of the plasma membrane; however, it is possible that GPI anchors could promote clustering of PME and PMEI pairs to fine-tune pectin structure in response to developmental or stress cues (Braybrook and Peaucelle 2013; Wu et al. 2018).

Other pectin-modifying enzymes are also predicted to be GPI anchored. Pectin lyases cleave the homogalacturonan backbone of pectin, preferentially acting on regions that have been demethylesterified by the action of PMEs (Wang et al. 2018). Pectin Lyase-Like 13 was initially identified as Powdery Mildew Resistance 6 (PMR6), with the *pmr6-1* phenotype displaying smaller rosette leaves, increased pectin content, and increased pectin demethylesterification (Vogel et al. 2002; Sun and van Nocker 2010). As for PMEI1, the requirement for GPI anchorage of PMR6 has not been demonstrated. Potential roles for the GPI anchor of PMR6 would include contributing to the localized release of pectin hydrolysis products that act as signaling intermediates (Hernandez-Blanco et al. 2007). The small size of *pmr6-1* plants implicates PMR6 in normal growth and development, although the function and requirement of the putative GPI anchor warrants further investigation.

### GPI-anchored cell wall sensors

An important function of plant GPI-anchored proteins is to support the transduction of signals regarding the status of the cell wall into the cell. This is typified by the interaction of 2 plant GPI-anchored proteins, LORELEI (LRE) and LRE-like GPI-Anchored Protein 1 (LLG1), with the extensively studied Catharanthus roseus RLK1-like (CrRLK1L) receptor kinase FERONIA (FER) (Liu et al. 2016a, b; Feng et al. 2018). LRE and LLG1 interact directly with FER in the apoplast, activating distinct signaling cascades at different developmental stages and in response to changes in environmental conditions. The attachment of a GPI anchor to LRE is essential for its localization to the outer leaflet of the plasma membrane in the filiform apparatus but not for its function during pollen tube reception (Liu et al. 2016a, 2016b). FER is involved in sensing the softening of the cell wall under high salt conditions, triggering multiple calcium signaling pathways, including the release of Ca^2+^ into the wall to reinforce the cell wall matrix via pectin-Ca^2+^ crosslinking (Feng et al. 2018). Intriguingly, FER was recently revealed to directly bind to demethylesterified pectin in the cell wall and regulate signaling via Rho-like GTPases, Rho of plants (ROP) 2, 6, and 11. These ROPs are also S-acylated and prenylated (see below), implying that multiple lipid modifications are important for different steps in the plant cell wall integrity and stress signaling (Chai et al. 2016; Sugiyama et al. 2019; Lin et al. 2022a, 2022b, 2022c).

### COBRA and COBRA-like families

**Figure 3.**
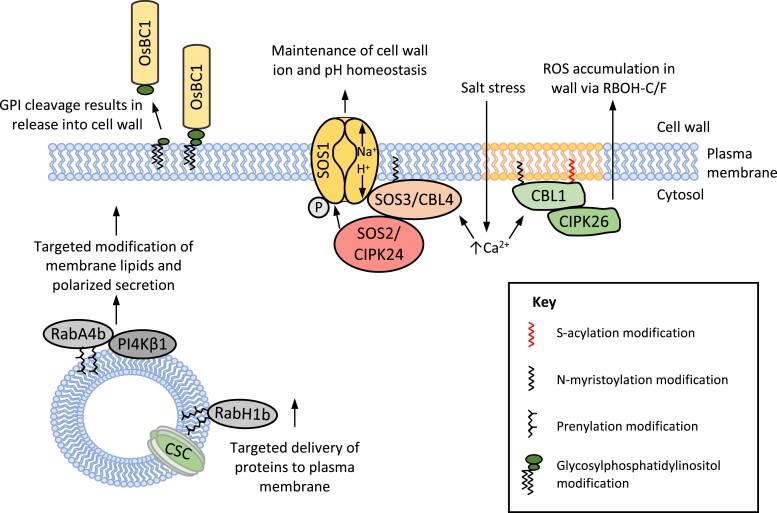
Lipid-modified proteins in cell wall integrity signaling, biosynthesis, homeostasis, and salt stress perception. Representative examples to illustrate the diverse roles of lipid-modified proteins in regulating cell wall properties and signaling. The prenylated Rab GTPases, RabA4b and RabH1b, coordinate the trafficking of PI4Kβ1 and the CSC, respectively. A rice COBRA-like protein, OsBC1 has a cleavable GPI anchor, allowing its release into the cell wall. Increased cytosolic Ca^2+^ acts as a second messenger during salt stress and with N-myristoylation promotes the plasma membrane localization of SALT OVERLY SENSITIVE 3/CALCINEURIN B-LIKE PROTEIN 4 (SOS3/CBL4) and for interaction and activation of Na+/H+ antiporter SOS1 by SOS2/CBL-INTERACTING PROTEIN KINASE 24 (CIPK24). CBL1 requires dual lipid modification with N-myristoylation and S-acylation for localization to membrane microdomains (yellow section of plasma membrane) and subsequent activation of CIPK26, promoting reactive oxygen species accumulation in the cell wall via RESPIRATORY BURST OXIDASE HOMOLOG PROTEINS C and F (RBOH-C/F) contributing to cell wall loosening.

The plant-specific COBRA (COB) and COBRA-like (COBL) genes encode GPI-anchored proteins that are essential for cell wall biosynthesis, regulating cellulose crystallinity and contributing to cell wall expansion and anisotropic growth (Roudier et al. 2002; Brady et al. 2007). The founding member of the COB family was identified due to its mutant displaying abnormal root expansion and reduced crystalline cellulose with increased amounts of disorganized cellulose (Benfey et al. 1993; Schindelman et al. 2001). Many COB and COBL genes are coexpressed with cellulose biosynthesis genes, owing to their function in binding to cellulose during its deposition in the cell wall (Brown et al. 2005; Brady et al. 2007; Ben-Tov et al. 2018). Investigation of COB and COBL function has been carried out extensively in crop species. Rice (*Oryza sativa*) BRITTLE CULM 1 (OsBC1), a homologue of COBL4, exhibits abnormal secondary cell wall deposition with reduced cellulose and increased lignin content, resulting in their characteristic brittle culms (Sato-Izawa et al. 2020). OsBC1 interacts with crystalline cellulose through its carbohydrate-binding domain and is anchored in the plasma membrane by its GPI anchor (Liu et al. 2013). The GPI anchor can be cleaved to release OsBC1 into the cell wall; however, the significance of this cleavage is still unclear (Fig. 3) (Liu et al. 2013). Mutation of OsBC16, involved in GPI lipid anchor synthesis, results in the same reduced cell wall elasticity, fragile internodes, and reduced cellulose content as *OsBC1* and is essential for GPI modification of OsBC1. Loss of OsBC16 also disrupts OsBC1 targeting to the plasma membrane and membrane nanodomains, which is likely important for its interaction with cellulose in the cell wall and implicates GPI anchoring in OsBC1 function (Xu et al. 2022a, 2022b). Further evidence of the importance of the GPI anchor in targeting proteins to particular regions of the plasma membrane comes from COBL10. GPI anchorage of COBL10 is essential for its localization to the apical tip of pollen tubes, where it functions in cell wall organization, likely by regulating the pectin matrix and cellulose orientation required for female-signal guided expansion of the pollen tube (Li et al. 2013). Genetic analysis has identified an essential role for other COBL proteins in diverse cell wall types. Mutation in *roothairless* 3 (*rth3*), a maize COBL gene family member, disrupts cell wall deposition during root hair development (Hochholdinger et al. 2008; Li et al. 2022), and *cobl*2 has defective seed coat mucilage polysaccharide organization (Ben-Tov et al. 2018).

### GPI-anchored proteins as coordinators of multicellular growth

The embryo-lethal *pnt1* mutant (see above) results from a defect in the synthesis of the GPI anchor (Gillmor et al. 2005). While embryos exhibit clear cell wall defects, callus derived from the mutant exhibits none of these defects. This observation led the authors to suggest that proteins with GPI anchors may be particularly important in coordinating multicellular growth. Some support for this suggestion comes from studies on mutants in several GPI-anchored proteins. Mutants in both ZERZAUST, a protein with homology to β-1,3 endoglucanases, and SKU5 cause the twisting of organs, consistent with defective coordination between cell layers (Fulton et al. 2009; Vaddepalli et al. 2017). The triple mutant between *SKU5* and two related GPI-anchored proteins SKU5-SIMILAR1 (SKS1) and SKS3 exhibit huge variation in cell size even between adjacent cells (Sedbrook et al. 2002; Zhou 2019). It is unclear how these GPI-anchored proteins coordinate cell growth, but they do illustrate very clearly the link between proper cell wall synthesis and cell expansion. It is possible that GPI-anchored proteins, such as SKU5, generate signals that are perceived by adjacent cells. SKU5 is characterized by 3 domains exhibiting homology to blue copper oxidases (Fig. 2); however, it lacks several conserved amino acids likely to be essential for activity and consequently. Any enzymatic function and any signal this might generate remain unknown. More recently SKU5 and SKS1 have been shown to modulate ROS-based signals generated by NADPH oxidase, and this modulation is essential for cell wall structure and root growth. The GPI anchor was absolutely essential for the function and targeting of SKU5 and SKS1 to the plasma membrane (Chen et al. 2023).

Unfortunately, due to space limitations, we could not include the details of many other genes encoding GPI-anchored proteins with novel functions in the cell wall. Other examples of GPI-anchored proteins with known or putative roles in cell wall biosynthesis and regulation of which have also recently been reviewed (Zhou 2022).

## N-myristoylation and the cell wall

Protein N-myristoylation is the addition of a saturated 14-carbon fatty acid moiety to the alpha-amino group of the N-terminal glycine residue of a protein after the removal of the initiator methionine by a methionine aminopeptidase (Giglione et al. 2000). It is also possible that myristoylation occurs at internal glycine residues following protein cleavage that reveals a newly exposed terminal glycine, as has been shown for human proteins (Thinon et al. 2014; Majeran et al. 2018). The myristate group is added by the formation of an amide linkage to the glycine (Fig. 1), and currently, there is no evidence that the myristate group is removed in plants. However, the myristoyl group can be masked in various ways to regulate its membrane association (Matheson et al. 2008; Giglione and Meinnel 2022). N-myristoylation supports membrane interaction but is not sufficient for stable membrane association and therefore acts as a substrate for further lipid modifications such as S-acylation by membrane-bound DHHC-type protein acyl transferases (PATs) (Batistic 2012; Castrec et al. 2018). In Arabidopsis, there are 2 N-myristoyltransferases (NMTs), and knockout of the more active, NMT1, is lethal, whereas *nmt2* mutants display defects in the shoot apical meristem during embryonic development, aberrant cell polarity, and growth arrest, implicating N-myristoylation in multiple developmental processes that require appropriate cell wall maintenance and deposition (Pierre et al. 2007; Renna et al. 2013).

### Myristoylation of SOS3 for cell wall integrity maintenance during salt stress

Perception of salt stress is important for plants to mount appropriate responses to maintain their growth and development. Sensing salt concentrations occurs at the cell wall, and signals generated in the wall are transduced into the cell via plasma membrane-localized receptors, such as described for GPI-anchored proteins FLA4 and LRE/LLG1. Salt overly sensitive 3/calcineurin B-like protein (SOS3/CBL4) is an N-myristoylated Ca^2+^ sensor that activates SOS2, a protein kinase required for salt tolerance by maintaining normal Na^+^/K^+^ concentration within the cell (Liu et al. 2000). Together SOS2/3 activate the Na^+^/H^+^ antiporter, SOS1, at the plasma membrane, resulting in the transport of Na^+^ out of the cell. N-myristoylation of SOS3 is required for the calcium-dependent interaction of SOS3 with SOS2 and is also required for salt tolerance (Fig. 3) (Mahajan et al. 2008). Here, the coupling of Na^+^ export from the cytosol and H^+^ import could contribute to the maintenance of cell wall pH to prevent acidification-related loosening of the cell wall by expansins, thus maintaining cell wall integrity (Cosgrove 2016; Colin et al. 2023). Similarly, extracellular pH is also coordinated with cellulose synthesis during cell expansion by the GPI-anchored protein SHAVEN3 and its homolog, SHAVEN3-like 1 (Hayashi et al. 2008; Yeats et al. 2016).

### Maintenance of polarized cell wall deposition at root hair tip by CBLs

Root hairs are long and thin extensions of root epidermal cells, and their formation requires the polarized extension of the cell by deposition of new cell wall material at the apex of the elongating root hair. This extension requires apoplastic accumulation of reactive oxygen species (ROS) and Ca^2+^ gradients for the appropriate cell wall loosening for expansion and for the localized secretion of cell wall components (Zhang et al. 2022). Like SOS3/CBL4, other CBLs, CBL1, 5, and 9, are dual-lipid modified with N-myristoylation and S-acylation. Dual lipid modification is required for CBL1 localization to the plasma membrane (Batistic et al. 2008). CBL1 contributes to the membrane lipid environment, Ca^2+^ gradients, and ROS production, all of which are required for the polar delivery of cell wall components at the tip of root hairs (Fig. 3) (Preuss et al. 2006; Zhang et al. 2022).

## Prenylated proteins at the plant cell wall

Prenylation is the irreversible addition of polyisoprene lipids to cysteine residues at the C terminus of proteins; typically 15-carbon farnesyl or 20-carbon geranylgeranyl units are added (Fig. 1). Like other lipid modifications, prenylation introduces a hydrophobic group that contributes to the interaction of the modified protein with specific cellular membranes. Farnesyl and geranylgeranyl moieties are added through thioether linkages by protein farnesyl transferase (PFT) and protein geranylgeranyl transferase (PGGT-I). These prenyltransferases are heterodimeric, with a common α subunit and β subunits that confer protein specificity and determine modification by farnesyl or geranylgeranyl lipid (Hemsley 2015). Prenylation by PFT and PGGT-I occurs at the cysteine residue of the CaaX box consensus at the protein C terminus, where “a” represents aliphatic amino acids and “X” represents any amino acid that depends on the substrate. Following prenylation, the aaX peptide is removed, and the newly freed carboxyl group of the new terminal cysteine is methylated (Fig. 1) (Chang et al. 2021).

### Dual geranylgeranyl modification of Rab GTPases

In addition to PFT and PGGT-I, there exists a Rab GTPase-specific geranylgeranyl transferase, RabGGT, that appears to modify only Rab family members, acting only on Rab GTPases in complex with Rab Escort Protein (REP). Twin geranylgeranyl modification of Rab GTPases is less reliant on the CaaX motif, and instead, RabGGT specificity is imposed by interaction with REP, prenylating both cysteines at XCC, XCXC, XCCX, CCXX, and CCXXX motifs (Shi et al. 2016). Rabs are small GTPases involved in vesicular transport and function in many plant-specific signaling pathways involving cell wall regulation and pathogen defense (Gutkowska et al. 2021). Many of the components of the cell wall, such as hemicelluloses and pectins, are synthesized by enzymes in the endomembrane system before their eventual targeted release into the cell wall (Kim and Brandizzi 2014; Meents et al. 2019). Membrane cycling of Rab GTPases is critical to their interaction with their respective effectors and the resulting appropriate vesicular transport implicating them in multiple cell wall-related processes (MartiniÈre and Moreau 2020). In Arabidopsis, global disruption of Rab geranylgeranyl modification by mutation of the *RGTB1* gene, encoding the RGTβ subunit, results in pollen tube and root hair defects, reminiscent of those seen for mutants of N-myristoylated and S-acylated CBL1 (Batistic et al. 2008; Gutkowska et al. 2015). This is likely a result of defects in the endocytic and exocytic vesicular trafficking needed to maintain polarized cell expansion at tip-growing cells (van de Meene et al. 2017).

Cellulose synthesis is partially regulated by trafficking cellulose synthase complexes (CSCs) to and from the plasma membrane, where they are active in cellulose biosynthesis (Zhu and McFarlane 2022). RabH1b has recently been implicated in this cycling whereby disruption of the *RabH1b* gene results in reduced motility of the primary cell wall CELLULOSE SYNTHASE A6 at the plasma membrane (Fig. 3) (He et al. 2018). Although RabH1b is not currently confirmed to be prenylated, it does have appropriately situated cysteines for recognition by REP. Similarly, RabA4b is putatively prenylated and is identified as an interactor of phosphatidylinositol 4-OH kinase, PI4Kβ1, potentially implicating it in the regulation of membrane lipid at the trans-Golgi network (Fig. 3) (Preuss et al. 2006; Jia et al. 2018; Zhou et al. 2020). The role of the RabA4 subfamily in tip-growing cells is also illustrated by the requirement for RabA4d for pollen tube growth (Szumlanski and Nielsen 2009). Overall, Rab GTPases require prenylation for their membrane interaction and function and even have their own prenyltransferases. However, it is still unclear whether the phenotypes associated with Rab mutants are due to global defects in vesicular transport or can be attributed directly to the transport of cell wall related enzymes and cargoes.

### Prenylated ROPs

In the moss *Physcomitrella patens*, knockout of the prenyltransferases *PpGGTβ* subunit has been implicated in cell-cell adhesion through the maintenance of cell wall integrity that is required to maintain multicellularity (Bao et al. 2022). The *PpGGTβ* loss of multicellularity and polar cell elongation phenotype was rescued by the introduction of the Arabidopsis PGGT-I, indicating that the geranylgeranyl transferase function is conserved (Thole et al. 2014). The phenotype of *PpGGTβ* has since been linked to the geranylgeranyl modification of ROP GTPases (Bao et al. 2022). Indeed, ROP involvement in cell wall biosynthesis is well documented in root hair growth, cell wall status monitoring, and secondary cell wall patterning through the regulation of microtubule dynamics (Chai et al. 2016; Feiguelman et al. 2022; Lin et al. 2022a, 2022b, 2022c; Xu et al. 2022a, 2022b). Type II ROPs (ROP9, 10, and 11) are S-acylated, and Type I ROPs (ROP2, 4, and 6) are prenylated, although some type I ROPs are dual modified, bearing both prenylation and S-acylation (Li et al. 2022).

## S-acylation of cell wall–related proteins

S-acylation, or palmitoylation, as it is commonly known, is the post-translational addition of a fatty acid moiety, usually stearate or palmitate in plants, to a cysteine residue (reviewed in Chamberlain and Shipston 2015; Hurst and Hemsley 2015; Hemsley 2017; Li and Qi 2017; Zheng et al. 2019; Hemsley 2020; Wang and Yang 2021; Li et al. 2022). The hydrophobic nature of the fatty acid molecules means that S-acylation, like other lipid modifications of proteins, increases the membrane affinity of the modified proteins. For cytosolic proteins, S-acylation results in membrane association; however, S-acylation is also known to occur in proteins that possess transmembrane helices. For these transmembrane proteins, the roles of S-acylation include protein targeting to specific membrane microdomains, protein trafficking, protein-protein interactions, tilting of trans-membrane helices, and protein stability (Charollais and Van Der Goot 2009; Chamberlain and Shipston 2015). Unlike other lipid modifications of proteins, S-acylation is freely reversible.

Several hundred plant proteins were identified to be S-acylated using global proteomic approaches (Hemsley et al. 2013; Srivastava et al. 2016). However, further progress was hampered due to a lack of information on the exact sites of S-acylation. This knowledge gap was addressed in a recent study that identified 1849 S-acylated cysteines located within 1094 proteins and estimated that around 6% of all proteins are modified by S-acylation (Kumar et al. 2022). Although S-acylated proteins are involved in many aspects of plant metabolism and signaling, modification by S-acylation features prominently among proteins involved in cell wall biosynthesis and cellulose synthesis in particular.

### Cellulose synthesis

**Figure 4.**
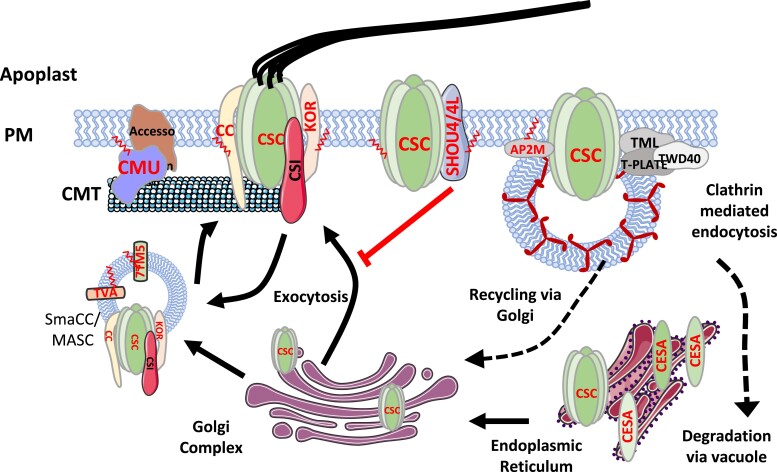
S-acylated proteins during CSC assembly and trafficking. CELLULOSE SYNTHASE A (CESA) proteins form the core of the CSC. A host of other proteins are known to either: colocalize with the CSC, associated with assembly and trafficking of the CSC to the plasma membrane (PM) or facilitate interactions between the cytoskeleton, the CSC and/or PM (reviewed in (Lampugnani et al. 2019; Polko and Kieber 2019; Gu and Rasmussen 2022). Many of these proteins are modified by S-acylation (indicated by red text). Proteins involved in cellulose synthesis illustrate the varied biological roles that S-acylation can play in protein function like membrane anchoring, subcellular trafficking and aiding the formation of protein complexes. S-acyl groups are shown for illustrative purposes and are indicated by a red zigzag line. The PM and cortical microtubule (CMT) are indicated together with proteins involved in cellulose synthesis of trafficking of the CSC. CMU, TPLATE Complex MUNISCIN- LIKE (TML), TRANSDUCIN/WD40 (TWD40), CSI, TARNVIA (TVA), MICROTUBULE-ASSOCIATED CELLULOSE SYNTHASE COMPARTMENT/Small CESA Compartments (MASC/SmaCC), KORRIGAN (KOR), TRANSMEMBRANE DOMAIN 5 (7TM5), ADAPTER PROTEIN COMPLEX 2 (AP2M), and COMPANION OF CELLULOSE SYNTHASE (CC) are indicated.

CELLULOSE SYNTHASE A (CESA) proteins, the catalytic subunits of the CSC, are among the most heavily modified proteins by S-acylation, with up to 8 cysteines modified in different CESA proteins. In Arabidopsis CESA7, 4 cysteines in a region known as variable region 2 (VR2) and 2 cysteines in the C-terminus (CT) are modified by S-acylation, and these are important for plasma membrane localization of the CSC (Kumar et al. 2016). A subsequent global proteomic study confirmed that VR2 and/or CT cysteines are S-acylated in both primary and secondary cell wall CESA proteins (Kumar et al. 2022). However, sites outside of these regions were also S-acylated in some CESA proteins, pointing to functional diversification of some protein domains between different CESA proteins. In particular, the cysteines located toward the amino terminus, within a putative RING finger domain and assumed to chelate zinc, were identified to be S-acylated for CESA8. Modification of these cysteines by S-acylation is not compatible with a role in zinc binding. These data suggest that, although the RING domain of all CESA proteins contain 8 conserved cysteines, there is structural, and probably functional, divergence between the domains from CESA7/CESA4/CESA8. Many other proteins involved in cellulose synthesis were also identified as being S-acylated (Fig. 4) (Kumar et al. 2022). These include proteins that colocalize with the CSC, such as KORRIGAN (Vain et al. 2014), COMPANION OF CELLULOSE SYNTHASE 1 (Endler et al. 2015), and CELLULOSE SYNTHASE INTERACTING (CSI) (Gu et al. 2010); proteins that mediate CSC trafficking, such as SHOU4, SHOU4L (Polko et al. 2018), TRANVIA (Vellosillo et al. 2021), and 7TM5 (McFarlane et al. 2021); in addition to proteins that are involved in exocytosis or endocytosis of the CSC, such as AP2M (Bashline et al. 2013) and EXO84B (Gu and Rasmussen 2022). The function of S-acylation of these proteins is not entirely clear; however, it is known that S-acylation can promote the clustering of membrane proteins. The highly modified nature of the CSC and the very hydrophobic environment this creates may mean that only proteins that are S-acylated and similarly hydrophobic are able to associate with the CSC.

Microtubules play a crucial role during cellulose synthesis, where they are involved in both the delivery of CSCs to the plasma membrane and guiding CSC movement through the plasma membrane. Tubulin proteins, the structural components of microtubules; chaperonin containing T-complex polypeptide-1 complex, a multi-subunit chaperonin complex that is involved in tubulin folding (Ahn et al. 2019); and CELLULOSE MICROTUBULE UNCOUPLING (CMU) proteins that anchor the cortical microtubules to the plasma membrane (PM) (Liu et al. 2016a, 2016b) are all modified by S-acylation (Hemsley et al. 2008; Kumar et al. 2022). Although none of these proteins contain transmembrane helices, some, like CMU, are known to localize to membranes. It is likely that S-acylation will contribute to their membrane localization and probably their association with sites of cellulose synthesis.

### Matrix polysaccharides

Proteins involved in the biosynthesis of other major cell wall polysaccharides, hemicelluloses and pectins are also modified by S-acylation (Kumar et al. 2022). Compared with cellulose biosynthesis, far fewer biosynthetic proteins for noncellulosic polysaccharides are modified by S-acylation. For example, among the xylan biosynthesis proteins, only GlucUronic acid substitution of Xylan 1, which is involved in the addition of glucuronic acid and 4-O-methyl glucuronic acid side-chains to xylan (Mortimer et al. 2010), is S-acylated (Kumar et al. 2022). Similarly, for xyloglucan biosynthesis, only MUR3, a galactosyltransferase that acts specifically on the third xylose residue within the XXXG core structure of xyloglucan (Madson et al. 2003), is S-acylated. Arabidopsis MUCILAGE-RELATED10 (MUCI10) and closely related paralog GLYCOSYLTRANSFERASE 6, are both S-acylated. MUCI10 has been proposed to be involved in the decoration of glucomannan chains (Voiniciuc et al. 2015), which are synthesized by the action of CELLULOSE SYNTHASE-LIKE A2. COTTON GOLGI-RELATED 2 (CGR2) and CGR3 play an important role in the methylesterification of pectins (Kim et al. 2015), and both proteins are S-acylated. It is interesting to note that all hemicellulose and pectin biosynthetic proteins that are S-acylated act on the side chains of the polysaccharides rather than the backbone. It is possible that this may reflect the organization of hemicellulose and pectin biosynthesis pathways within the Golgi with S-acylation targeting proteins to where they are best localized to affect later steps in the biosynthetic pathway.

Additionally, several hemicellulose and pectin modification and degradation enzymes are also S-acylated. This includes XYLANASE1, which is involved in xylan degradation; BETA-GALACTOSIDASE 10, xyloglucan endotransglucosylase/hydrolases XTH31 and XTH32 involved in xyloglucan modification and degradation; and a number of proteins that are involved in pectin modification and degradation like PECTIN METHYLESTERASE 16 (PME16), PME inhibitor PMEI-PME51, beta galactosidases BGAL1, BGAL2, BGAL6, BGAL8, BGAL9, beta-D-xylosidases BXL4, BXL6, and pectin acetylesterases PAE5, PAE7, PAE8 and PAE12 (Kumar et al. 2022). All of these enzymes are localized to the apoplast, so the presumed role of S-acylation for these enzymes would be in their trafficking to the extracellular space via exocytosis.

### Lignin biosynthesis

Biosynthesis of monolignols, the building blocks of lignin, takes place at the interface of the cytosol and ER membrane. Several enzymes catalyzing the formation of monolignol subunits are modified by S-acylation, including PHENYLALANINE AMMONIA-LYASE, 4-COUMARATE:COA LIGASE, CAFFEOYL COENZYME A ESTER O-METHYLTRANSFERASE, CINNAMOYL COA REDUCTASE, CAFFEATE O-METHYLTRANSFERASE, and CINNAMYL ALCOHOL DEHYDROGENASE (Kumar et al. 2022). None of these monolignol biosynthesis enzymes possess a transmembrane helix, and therefore S-acylation could provide a mechanism for the transient anchoring of these enzymes to ER membranes.

### Cell wall integrity sensing

Cell wall integrity–sensing mechanisms involve protein sensors that detect cell wall damage or other mechanical cues and activate the downstream signaling events. FEI1 is one such sensor that belongs to the Leucine-rich repeat receptor-like kinase family and is plasma membrane localized. FEI1 interacts with the GPI-anchored protein FLA4 but is also S-acylated (Kumar et al. 2022). S-acylation of a receptor kinase, FLS2, has recently been shown to be important for receptor complex activation in response to the binding of its ligand (Hurst et al. 2023), and S-acylation may serve a similar role with FEI1. Whether S-acylation may target FEI1 to membrane domains where it is able to colocalize with GPI-anchored proteins in the same pathway is unclear. MID1-COMPLEMENTING ACTIVITY1 (MCA1), a mechano-sensitive calcium channel (Nakagawa et al. 2007), is also S-acylated. MCA1 functions downstream of Theseus1, another integrity sensor that belongs to the CrRLK1L subfamily of protein kinases. MCA1 has been suggested to be involved in the production of jasmonic acid and salicylic acid in seedlings treated with the cellulose synthase inhibitor isoxaben (Denness et al. 2011; Engelsdorf et al. 2018).

### (De-)S-acylation enzymes and dynamics

S-acylation is a reversible modification with the addition of the fatty acid catalyzed by PATs and its removal by APTs. In Arabidopsis, there are 24 PAT enzymes that are localized to various subcellular spaces (Batistic 2012). In animal species, de-S-acylation is catalyzed by 1 to 2 serine hydrolases (APT1/2) (Dekker et al. 2010) and Alpha/Beta Hydrolase Domain-containing 17A/B/C (ABHD17) proteins (Lin and Conibear 2015). Although the homologs of APT1/2 have not been identified in plants, 11 members of the Arabidopsis ABHD17-like acyl protein thioesterases family were reported to exhibit thioesterase activity (Liu et al. 2021).

Cycling of a modified protein between S-acylated and de-S-acylated states, by the action of PATs and APTs, offers a powerful and dynamic regulatory mechanism for protein function. Although it is possible to see the effects of myristoylation by altering the protein structure to mask the modification, S-acylation is reversible in a manor analogous to that of other common protein modifications, such as phosphorylation (Wang et al. 2017).

Although future studies are likely to reveal more information on how the reversibility of lipid modification is important for proteins cycling between the plasma membrane and the cytosol, current examples of (de-)S-acylation enzyme/substrate pairs or dynamic regulation of S-acylation are rare in plants. One notable exception is SGN1, a receptor-like cytoplasmic kinase (RLCK) that belongs to the RLCK VII family. It is required for proper positioning and formation of Casparian strip (Alassimone et al. 2016). Although SGN1 is localized to the plasma membrane, it does not contain any TM helices with S-acylation being responsible for its plasma membrane attachment. In the presence of the S-acylation inhibitor, 2-bromo-palmitate, SGN1 accumulates in the cytosol, even if the samples have been pre-treated with protein synthesis inhibitor, cycloheximide. These observations suggested that SGN1 undergoes cycles of S-acylation and de-S-acylation resulting in repeated attachment and de-attachment to the plasma membrane, helping establish polarity of SGN1 and consequent proper localization of the Casparian strip within the cell wall (Alassimone et al. 2016).

### S-acylation perspectives

Identification of an S-acylated protein and the potential cysteines at which it might be modified is only the first step in the investigation of the role of S-acylation in the function of a particular protein and the biological process it is involved in. This is followed by mutation of target cysteines and studying the effect of loss of S-acylation on the cell biology and/or biochemistry of the target protein, thereby establishing a more precise role of S-acylation in a given process. Among the cell wall–related proteins, so far, this kind of analysis has only been performed for a small number of proteins like CESA proteins (Kumar et al. 2016) and KORRIGAN (Kumar et al. 2022). The availability of the comprehensive acylome dataset with the acyl-site information has the potential to greatly facilitate such studies.

It is important to establish a full complement of substrates for the PATs and the APTs. Given that there are more than 1000 S-acylated proteins and only 24 PATs and 11 APTs, each of the PAT and APT is likely to act on dozens, if not hundreds, of substrate proteins. Although PAT/APT substrates have been identified (Liu et al. 2021), further studies involving novel technologies, such as proximity labelling and high throughput proteomics, will be required to establish the identity of those substrates and untangle the function of individual PAT and APT enzymes. The ultimate goal would be to establish the regulatory network where all components involved in S-acylation and de-S-acylation of a target protein are known. Only then will we be able to establish how S-acylation modifies protein function in response to endogenous and exogenous cues.

## Concluding remarks

The post-translational modification of proteins with a lipid moiety has functions in protein-protein interaction, targeting proteins to specific membranes and regions within membranes. Through these functions, lipid-modified proteins have roles in communicating the cell wall status into the cell as well as controlling normal cell wall deposition during development and in response to biotic and abiotic stress. N-myristoylation, prenylation, and S-acylation are involved in many similar functions. In some instances, this is not surprising because both prenylation and N-myristoylation can precede modification by S-acylation (see above), although these modifications involve proteins localizing to the endomembrane and the inner leaflet of the plasma membrane. In contrast, GPI-anchors tether proteins to the plasma membrane but outside of the cell. Both S-acylation and GPI-anchors have the potential to support the clustering of cell wall synthesis enzymes and integrity receptors. However, they operate on opposite sides of the plasma membrane. Whether these modifications function entirely independently is unclear because there is no information regarding any similarities between protein clustering driven by S-acylation or the addition of a GPI-anchor. The potential for inter-leaflet communication and organization of proteins within both leaflets of the plasma membrane would be an interesting avenue for future research.

An increasingly large number of GPI-anchored proteins have been identified as being essential for normal cell wall deposition. Many of these proteins share little in common other than the GPI anchor. In many cases, it remains unknown how they function to alter the cell wall (see “Outstanding Questions”). The fact that these GPI-anchored proteins are likely to localize on the outer leaflet of the plasma membrane highlights the importance of the environment in the region immediately adjacent to the plasma membrane as being of central importance to normal cell wall deposition, but one that is very sensitive to external signals and perturbations. GPI-anchored proteins that apparently regulate cell wall deposition and integrity sensing, and coordinate multicellular growth, reflect the fact that cell wall deposition must be sensitive to feedback from inside the cell, the cell wall, adjacent cells, and potential biotic interactions. A diverse set of GPI-anchored proteins are important for all these processes (Fig. 2), and the GPI anchor is responsible for their targetting to the outer leaflet of the plasma membrane, where they are best positioned to perceive these signals and interact with other cell surface receptors as well as components of the cell wall biosynthesis machinery such as the CSC.

Although N-myristoylated, prenylated, and S-acylated proteins also exhibit lipid/fatty acid modification, they share very little in common with GPI-anchored proteins with the former having the attached lipid group and at least part of the protein remaining within the cell. The role of prenylated and N-myristoylated proteins in cell wall deposition is limited. However, these modifications have a well-characterized role in modifying small GTPases. These small GTPases are important for polarized vesicle targeting and are consequently particularly important in processes such as tip growth. Given that around 6% of proteins are modified by S-acylation, it is not surprising that some proteins related to cell wall biosynthesis are modified in this way, however, the fact that most if not all proteins related to cellulose synthesis are modified in this way and the particularly large number of acyl groups on the CSC suggests that it plays a particularly important role in cellulose synthesis. S-acylation is known to promote protein-protein interactions, and potentially this may be its role in the cell. Whether this is the result of the CSC localizing in specialized membrane domains is unknown. S-acylation of other proteins such as those involved in matrix polysaccharide or lignin biosynthesis may also promote the formation of enzyme complexes involved in substrate channeling (see “Outstanding Questions”).

ADVANCES BOX

- Genetic analysis has identified many diverse GPI-anchored proteins that play crucial roles in cell wall deposition during growth. These proteins include those that maintain a suitable environment in the apoplast for normal growth.
- The role of N-myristoylation and prenylation in cell wall biosynthesis is limited to the well-established functions of small GTPases that are important for polar secretion including that of cell wall components.
- Identification of an acylome facilitates the functional analysis of protein S-acylation by identifying which cysteines to mutate.
- Most if not all proteins involved in cellulose synthesis and the association of cellulose synthesis with microtubules are S-acylated, in contrast, only a small proportion of proteins involved in the synthesis of matrix polysaccharides are modified in this way.
- Several potential cell wall modifying enzymes are S-acylated, which likely regulates their localization.

OUTSTANDING QUESTIONS BOX

- To what extent does the structure of GPI anchors vary, and is variation in the structure of the GPI anchor important in the localization of GPI-anchored proteins within the plasma membrane and therefore cell wall?
- How do GPI-anchored proteins function to coordinate multicellular growth?
- Why are so many structurally diverse GPI-anchored proteins important for normal cell wall synthesis during growth?
- Why is the targeting of GPI-anchored proteins to membrane domains important for their function?
- Does the S-acylation of cell wall biosynthesis proteins promote their interaction during cellulose synthesis?
- Does S-acylation promote enzyme association in the ER and substrate channeling during monolignol biosynthesis?
- Is the reversible nature of S-acylation important for cell wall biosynthesis and could this regulate proteins that control the secretion of cell wall modifying enzymes?

## Dive Curated Terms

The following phenotypic, genotypic, and functional terms are of significance to the work described in this paper:

SKU5 Gramene: AT4G12420
SKU5 Araport: AT4G12420

FLA11 Gramene: AT5G03170
FLA11 Araport: AT5G03170

PMR6 Gramene: AT3G54920
PMR6 Araport: AT3G54920

SKS1 Gramene: AT4G25240
SKS1 Araport: AT4G25240

SOS3 Gramene: AT5G24270
SOS3 Araport: AT5G24270

COBL10 Gramene: AT3G20580
COBL10 Araport: AT3G20580

FLA3 Gramene: AT2G24450
FLA3 Araport: AT2G24450

PMEI1 Gramene: AT1G48020
PMEI1 Araport: AT1G48020

SOS2 Gramene: AT5G35410
SOS2 Araport: AT5G35410

BGAL1 Gramene: AT3G13750
BGAL1 Araport: AT3G13750

FEI1 Gramene: AT1G31420
FEI1 Araport: AT1G31420

FEI2 Gramene: AT2G35620
FEI2 Araport: AT2G35620

MUR3 Gramene: AT2G20370
MUR3 Araport: AT2G20370

pme1 Gramene: AT4G12390
pme1 Araport: AT4G12390

NMT2 Gramene: AT1g48600
NMT2 Araport: AT1g48600

CESA Gramene: Cellulose synthase
CESA Araport: Cellulose synthase

## References

[kiad491-B1] Adebali O , Ortega DR, Zhulin IB. CDvist: a webserver for identification and visualization of conserved domains in protein sequences. Bioinformatics. 2015:31(9):1475–1477. 10.1093/bioinformatics/btu836 25527097 PMC4410658

[kiad491-B2] Ahn HK , Yoon JT, Choi I, Kim S, Lee HS, Pai HS. Functional characterization of chaperonin containing T-complex polypeptide-1 and its conserved and novel substrates in Arabidopsis. J Exp Bot. 2019:70(10):2741–2757. 10.1093/jxb/erz099 30825377 PMC6506772

[kiad491-B3] Alassimone J , Fujita S, Doblas VG, van Dop M, Barberon M, Kalmbach L, Vermeer JEM, Rojas-Murcia N, Santuari L, Hardtke CS, et al Polarly localized kinase SGN1 is required for Casparian strip integrity and positioning. Nat Plants. 2016:2:16113. 10.1038/nplants.2016.113 27455051

[kiad491-B4] Bao L , Ren J, Nguyen M, Slusarczyk AS, Thole JM, Martinez SP, Huang J, Fujita T, Running MP. The cellular function of ROP GTPase prenylation is important for multicellularity in the moss Physcomitrium patens. Development. 2022:149(12):dev200279. 10.1242/dev.200279 35660859

[kiad491-B5] Bashline L , Li SD, Anderson CT, Lei L, Gu Y. The endocytosis of cellulose synthase in arabidopsis is dependent on mu 2, a clathrin-mediated endocytosis adaptin. Plant Physiol. 2013:163(1):150–160. 10.1104/pp.113.221234 23843604 PMC3762637

[kiad491-B6] Basu D , Tian L, Debrosse T, Poirier E, Emch K, Herock H, Travers A, Showalter AM. Glycosylation of a Fasciclin-like arabinogalactan-protein (SOS5) mediates root growth and seed mucilage adherence via a cell wall receptor-like kinase (FEI1/FEI2) pathway in arabidopsis. PLoS One. 2016:11(1):e0145092. 10.1371/journal.pone.0145092 26731606 PMC4701510

[kiad491-B7] Batistic O . Genomics and localization of the arabidopsis DHHC-cysteine-rich domain S-acyltransferase protein family. Plant Physiol. 2012:160(3):1597–1612. 10.1104/pp.112.203968 22968831 PMC3490592

[kiad491-B8] Batistic O , Sorek N, Schultke S, Yalovsky S, Kudla J. Dual fatty acyl modification determines the localization and plasma membrane targeting of CBL/CIPK Ca2+ signaling complexes in Arabidopsis. Plant Cell. 2008:20(5):1346–1362. 10.1105/tpc.108.058123 18502848 PMC2438452

[kiad491-B9] Bellincampi D , Cervone F, Lionetti V. Plant cell wall dynamics and wall-related susceptibility in plant-pathogen interactions. Front Plant Sci. 2014:5:288. 10.3389/fpls.2014.00228 24904623 PMC4036129

[kiad491-B10] Ben-Tov D , Idan-Molakandov A, Hugger A, Ben-Shlush I, Guenl M, Yang B, Usadel B, Harpaz-Saad S. The role of COBRA-LIKE 2 function, as part of the complex network of interacting pathways regulating Arabidopsis seed mucilage polysaccharide matrix organization. Plant J. 2018:94(3):497–512. 10.1111/tpj.13871 29446495

[kiad491-B11] Benfey PN , Linstead PJ, Roberts K, Schiefelbein JW, Hauser MT, Aeschbacher RA. Root development in arabidopsis—4 mutants with dramatically altered root morphogenesis. Development. 1993:119(1):57–70. 10.1242/dev.119.1.57 8275864

[kiad491-B12] Bernat-Silvestre C , Sanchez-Simarro J, Ma YX, Montero-Pau J, Johnson K, Aniento F, Marcote MJ. AtPGAP1 functions as a GPI inositol-deacylase required for efficient transport of GPI-anchored proteins. Plant Physiol. 2021:187(4):2156–2173. 10.1093/plphys/kiab384 34618080 PMC8644293

[kiad491-B13] Brady SM , Song S, Dhugga KS, Rafalski JA, Benfey PN. Combining expression and comparative evolutionary analysis. The COBRA gene family. Plant Physiol. 2007:143(1):172–187. 10.1104/pp.106.087262 17098858 PMC1761980

[kiad491-B14] Braybrook SA , Peaucelle A. Mechano-chemical aspects of organ formation in arabidopsis thaliana: the relationship between auxin and pectin. PLoS One. 2013:8(3):e57813. 10.1371/journal.pone.0057813 23554870 PMC3595255

[kiad491-B15] Brown DM , Zeef LAH, Ellis J, Goodacre R, Turner SR. Identification of novel genes in Arabidopsis involved in secondary cell wall formation using expression profiling and reverse genetics. Plant Cell. 2005:17(8):2281–2295. 10.1105/tpc.105.031542 15980264 PMC1182489

[kiad491-B16] Castrec B , Dian C, Ciccone S, Ebert CL, Bienvenut WV, Le Caer JP, Steyaert JM, Giglione C, Meinnel T. Structural and genomic decoding of human and plant myristoylomes reveals a definitive recognition pattern. Nat Chem Biol. 2018:14(7):671–679. 10.1038/s41589-018-0077-5 29892081

[kiad491-B17] Chai S , Ge F-R, Feng Q-N, Li S, Zhang Y. PLURIPETALA mediates ROP2 localization and stability in parallel to SCN1 but synergistically with TIP1 in root hairs. Plant J. 2016:86(5):413–425. 10.1111/tpj.13179 27037800

[kiad491-B18] Chamberlain LH , Shipston MJ. The physiology of protein S-acylation. Physiol Rev. 2015:95(2):341–376. 10.1152/physrev.00032.2014 25834228 PMC4551212

[kiad491-B19] Chang HY , Cheng TH, Wang AHJ. Structure, catalysis, and inhibition mechanism of prenyltransferase. IUBMB Life. 2021:73(1):40–63. 10.1002/iub.2418 33246356 PMC7839719

[kiad491-B20] Charollais J , Van Der Goot FG. Palmitoylation of membrane proteins (review). Mol Membr Biol. 2009:26(1):55–66. 10.1080/09687680802620369 19085289

[kiad491-B21] Chen C , Zhang Y, Cai J, Qiu Y, Li L, Gao C, Gao Y, Ke M, Wu S, Wei C, et al Multi-copper oxidases SKU5 and SKS1 coordinate cell wall formation using apoplastic redox-based reactions in roots. Plant Physiol. 2023:192(3):2243–2260. 10.1093/plphys/kiad207 37010107 PMC10315306

[kiad491-B22] Colin L , Ruhnow F, Zhu JK, Zhao CZ, Zhao Y, Persson S. The cell biology of primary cell walls during salt stress. Plant Cell. 2023:35(1):201–217. 10.1093/plcell/koac292 36149287 PMC9806596

[kiad491-B23] Cosgrove DJ . Growth of the plant cell wall. Nat Rev Mol Cell Biol. 2005:6(11):850–861. 10.1038/nrm1746 16261190

[kiad491-B24] Cosgrove DJ . Plant cell wall extensibility: connecting plant cell growth with cell wall structure, mechanics, and the action of wall-modifying enzymes. J Exp Bot. 2016:67(2):463–476. 10.1093/jxb/erv511 26608646

[kiad491-B25] De Caroli M , Lenucci MS, Di Sansebastiano GP, Dalessandro G, De Lorenzo G, Piro G. Protein trafficking to the cell wall occurs through mechanisms distinguishable from default sorting in tobacco. Plant J. 2011:65(2):295–308. 10.1111/j.1365-313X.2010.04421.x 21223393

[kiad491-B26] Dekker FJ , Rocks O, Vartak N, Menninger S, Hedberg C, Balamurugan R, Wetzel S, Renner S, Gerauer M, Scholermann B, et al Small-molecule inhibition of APT1 affects Ras localization and signaling. Nat Chem Biol. 2010:6(6):449–456. 10.1038/nchembio.362 20418879

[kiad491-B27] Denness L , McKenna JF, Segonzac C, Wormit A, Madhou P, Bennett M, Mansfield J, Zipfel C, Hamann T. Cell wall damage-induced lignin biosynthesis is regulated by a reactive oxygen species- and jasmonic acid-dependent process in arabidopsis. Plant Physiol. 2011:156(3):1364–1374. 10.1104/pp.111.175737 21546454 PMC3135913

[kiad491-B28] Desnoyer N , Palanivelu R. Bridging the GAPs in plant reproduction: a comparison of plant and animal GPI-anchored proteins. Plant Reprod. 2020:33(3–4):129–142. 10.1007/s00497-020-00395-9 32945906

[kiad491-B29] Endler A , Kesten C, Schneider R, Zhang Y, Ivakov A, Froehlich A, Funke N, Persson S. A mechanism for sustained cellulose synthesis during salt stress. Cell. 2015:162(6):1353–1364. 10.1016/j.cell.2015.08.028 26343580

[kiad491-B30] Engelsdorf T , Gigli-Bisceglia N, Veerabagu M, McKenna JF, Vaahtera L, Augstein F, Van der Does D, Zipfel C, Hamann T. The plant cell wall integrity maintenance and immune signaling systems cooperate to control stress responses in Arabidopsis thaliana. Sci Signal. 2018:11(536):eaao3070. 10.1126/scisignal.aao3070 29945884

[kiad491-B31] Feiguelman G , Cui X, Sternberg H, Ben Hur E, Higa T, Oda Y, Fu Y, Yalovsky S. Microtubule-associated ROP interactors affect microtubule dynamics and modulate cell wall patterning and root hair growth. Development. 2022:149(22):dev200811. 10.1242/dev.200811 36314989 PMC9845754

[kiad491-B32] Feng W , Kita D, Peaucelle A, Cartwright HN, Doan V, Duan QH, Liu MC, Maman J, Steinhorst L, Schmitz-Thom I, et al The FERONIA receptor kinase maintains cell-wall integrity during salt stress through Ca2+ signaling. Curr Biol. 2018:28(5):666–675. 10.1016/j.cub.2018.01.023 29456142 PMC5894116

[kiad491-B33] Fulton L , Batoux M, Vaddepalli P, Yadav RK, Busch W, Andersen SU, Jeong S, Lohmann JU, Schneitz K. DETORQUEO, QUIRKY, and ZERZAUST represent novel components involved in organ development mediated by the receptor-like kinase STRUBBELIG in Arabidopsis thaliana. PLoS Genet. 2009:5(1):e1000355. 10.1371/journal.pgen.1000355 19180193 PMC2628281

[kiad491-B34] Gigli-Bisceglia N , Engelsdorf T, Hamann T. Plant cell wall integrity maintenance in model plants and crop species-relevant cell wall components and underlying guiding principles. Cell Mol Life Sci. 2020:77(11):2049–2077. 10.1007/s00018-019-03388-8 31781810 PMC7256069

[kiad491-B35] Giglione C , Meinnel T. Mapping the myristoylome through a complete understanding of protein myristoylation biochemistry. Prog Lipid Res. 2022:85:101139. 10.1016/j.plipres.2021.101139 34793862

[kiad491-B36] Giglione C , Serero A, Pierre M, Boisson B, Meinnel T. Identification of eukaryotic peptide deformylases reveals universality of N-terminal protein processing mechanisms. EMBO J. 2000:19(21):5916–5929. 10.1093/emboj/19.21.5916 11060042 PMC305796

[kiad491-B37] Gillmor CS , Lukowitz W, Brininstool G, Sedbrook JC, Hamann T, Poindexter P, Somerville C. Glycosylphosphatidylinositol-anchored proteins are required for cell wall synthesis and morphogenesis in Arabidopsis. Plant Cell. 2005:17(4):1128–1140. 10.1105/tpc.105.031815 15772281 PMC1087991

[kiad491-B38] Gu Y , Kaplinsky N, Bringmann M, Cobb A, Carroll A, Sampathkumar A, Baskin TI, Persson S, Somerville CR. Identification of a cellulose synthase-associated protein required for cellulose biosynthesis. Proc Natl Acad Sci U S A. 2010:107(29):12866–12871. 10.1073/pnas.1007092107 20616083 PMC2919928

[kiad491-B39] Gu Y , Rasmussen CG. Cell biology of primary cell wall synthesis in plants. Plant Cell. 2022:34(1):103–128. 10.1093/plcell/koab249 34613413 PMC8774047

[kiad491-B40] Gutkowska M , Kaus-Drobek M, Hoffman-Sommer M, Pamula MM, Leja AD, Perycz M, Lichocka M, Witek A, Wojtas M, Dadlez M, et al Impact of C-terminal truncations in the Arabidopsis Rab escort protein (REP) on REP-Rab interaction and plant fertility. Plant J. 2021:108(5):1400–1421. 10.1111/tpj.15519 34592024 PMC9293207

[kiad491-B41] Gutkowska M , Wnuk M, Nowakowska J, Lichocka M, Stronkowski MM, Swiezewska E. Rab geranylgeranyl transferase beta subunit is essential for male fertility and tip growth in Arabidopsis. J Exp Bot. 2015:66(1):213–224. 10.1093/jxb/eru412 25316062 PMC4265159

[kiad491-B42] Hayashi S , Ishii T, Matsunaga T, Tominaga R, Kuromori T, Wada T, Shinozaki K, Hirayama T. The glycerophosphoryl diester phosphodiesterase-like proteins SHV3 and its homologs play important roles in cell wall organization. Plant Cell Physiol. 2008:49:1522–1535. 10.1093/pcp/pcn120 18718934

[kiad491-B43] He M , Lan M, Zhang B, Zhou Y, Wang Y, Zhu L, Yuan M, Fu Y. Rab-H1b is essential for trafficking of cellulose synthase and for hypocotyl growth in Arabidopsis thaliana. J Integr Plant Biol. 2018:60(11):1051–1069. 10.1111/jipb.12694 29975455

[kiad491-B44] He JD , Zhao H, Cheng ZL, Ke YW, Liu JX, Ma H. Evolution analysis of the Fasciclin-like arabinogalactan proteins in plants shows variable Fasciclin-AGP domain constitutions. Int J Mol Sci. 2019:20(8):1945. 10.3390/ijms20081945 31010036 PMC6514703

[kiad491-B45] Hemsley PA . The importance of lipid modified proteins in plants. New Phytol. 2015:205(2):476–489. 10.1111/nph.13085 25283240

[kiad491-B46] Hemsley PA . An outlook on protein S-acylation in plants: what are the next steps? J Exp Bot. 2017:68(12):3155–3164. 10.1093/jxb/erw497 28158736

[kiad491-B47] Hemsley PA . S-acylation in plants: an expanding field. Biochem Soc Trans. 2020:48(2):529–536. 10.1042/BST20190703 32239188

[kiad491-B48] Hemsley PA , Kemp AC, Grierson CS. The TIP GROWTH DEFECTIVE1 S-acyl transferase regulates plant cell growth in Arabidopsis. Plant Cell. 2005:17(9):2554–2563. 10.1105/tpc.105.031237 16100337 PMC1197434

[kiad491-B49] Hemsley PA , Taylor L, Grierson CS. Assaying protein palmitoylation in plants. Plant Methods. 2008:4:2. 10.1186/1746-4811-4-2 18190696 PMC2244627

[kiad491-B50] Hemsley PA , Weimar T, Lilley KS, Dupree P, Grierson CS. A proteomic approach identifies many novel palmitoylated proteins in Arabidopsis. New Phytol. 2013:197(3):805–814. 10.1111/nph.12077 23252521

[kiad491-B51] Hernandez-Blanco C , Feng DX, Hu J, Sanchez-Vallet A, Deslandes L, Llorente F, Berrocal-Lobo M, Keller H, Barlet X, Sanchez-Rodriguez C, et al Impairment of cellulose synthases required for Arabidopsis secondary cell wall formation enhances disease resistance. Plant Cell. 2007:19(3):890–903. 10.1105/tpc.106.048058 17351116 PMC1867366

[kiad491-B52] Hochholdinger F , Wen TJ, Zimmermann R, Chimot-Marolle P, Silva ODE, Bruce W, Lamkey KR, Wienand U, Schnable PS. The maize (Zea mays L.) roothairless3 gene encodes a putative GPI-anchored, monocot-specific, COBRA-like protein that significantly affects grain yield. Plant J. 2008:54(5):888–898. 10.1111/j.1365-313X.2008.03459.x 18298667 PMC2440564

[kiad491-B53] Houston K , Tucker MR, Chowdhury J, Shirley N, Little A. The plant cell wall: a complex and dynamic structure as revealed by the responses of genes under stress conditions. Front Plant Sci. 2016:7:984. 10.3389/fpls.2016.00984 27559336 PMC4978735

[kiad491-B54] Hurst CH , Hemsley PA. Current perspective on protein S-acylation in plants: more than just a fatty anchor? J Exp Bot. 2015:66(6):1599–1606. 10.1093/jxb/erv053 25725093

[kiad491-B55] Hurst CH , Turnbull D, Xhelilaj K, Myles S, Pflughaupt RL, Kopischke M, Davies P, Jones S, Robatzek S, Zipfel C, et al S-acylation stabilizes ligand-induced receptor kinase complex formation during plant pattern-triggered immune signaling. Curr Biol. 2023:33(8):1588–1596.e6. 10.1016/j.cub.2023.02.065 36924767

[kiad491-B56] Jia P-F , Xue Y, Li H-J, Yang W-C. Golgi-localized LOT regulates trans-Golgi network biogenesis and pollen tube growth. Proc Natl Acad Sci U S A. 2018:115(48):12307–12312. 10.1073/pnas.1809206115 30413616 PMC6275481

[kiad491-B57] Johnson KL , Jones BJ, Bacic A, Schultz CJ. The Fasciclin-like arabinogalactan proteins of Arabidopsis. A multigene family of putative cell adhesion molecules. Plant Physiol. 2003:133(4):1911–1925. 10.1104/pp.103.031237 14645732 PMC300743

[kiad491-B58] Kim SJ , Brandizzi F. The plant secretory pathway: an essential factory for building the plant cell wall. Plant Cell Physiol. 2014:55(4):687–693. 10.1093/pcp/pct197 24401957

[kiad491-B59] Kim SJ , Held MA, Zemelis S, Wilkerson C, Brandizzi F. CGR2 and CGR3 have critical overlapping roles in pectin methylesterification and plant growth in Arabidopsis thaliana. Plant J. 2015:82(2):208–220. 10.1111/tpj.12802 25704846

[kiad491-B60] Kinoshita T . Biosynthesis and biology of mammalian GPI-anchored proteins. Open Biol. 2020:10(3):190290. 10.1098/rsob.190290 32156170 PMC7125958

[kiad491-B61] Kinoshita T , Fujita M, Maeda Y. Biosynthesis, remodelling and functions of mammalian GPI-anchored proteins: recent progress. J Biochem. 2008:144(3):287–294. 10.1093/jb/mvn090 18635593

[kiad491-B62] Kumar M , CarrP, TurnerSR. An atlas of Arabidopsis protein S-acylation reveals its widespread role in plant cell organization and function. Nat Plants. 2022:8(6):670–681. 10.1038/s41477-022-01164-435681017

[kiad491-B63] Kumar M , Wightman R, Atanassov I, Gupta A, Hurst CH, Hemsley PA, Turner S. S-acylation of the cellulose synthase complex is essential for its plasma membrane localization. Science. 2016:353(6295):166–169. 10.1126/science.aaf4009 27387950

[kiad491-B64] Lalanne E , Honys D, Johnson A, Borner GHH, Lilley KS, Dupree P, Grossniklaus U, Twell D. SETH1 and SETH2, two components of the glycosylphosphatidylinositol anchor biosynthetic pathway, are required for pollen germination and tube growth in arabidopsis. Plant Cell. 2004:16(1):229–240. 10.1105/tpc.014407 14671020 PMC301407

[kiad491-B65] Lampugnani ER , Flores-Sandoval E, Tan QW, Mutwil M, Bowman JL, Persson S. Cellulose synthesis – central components and their evolutionary relationships. Trends Plant Sci. 2019:24:402–412. 10.1016/j.tplants.2019.02.011 30905522

[kiad491-B68] Li J , Yu MA, Geng LL, Zhao J. The Fasciclin-like arabinogalactan protein gene, FLA3, is involved in microspore development of Arabidopsis. Plant J. 2010:64(3):482–497. 10.1111/j.1365-313X.2010.04344.x 20807209

[kiad491-B69] Li JC , Zhang MQ, Zhou LJ. Protein S-acyltransferases and acyl protein thioesterases, regulation executors of protein S-acylation in plants. Front Plant Sci. 2022:13:956231. 10.3389/fpls.2022.866588 35968095 PMC9363829

[kiad491-B66] Li S , Ge F-R, Xu M, Zhao X-Y, Huang G-Q, Zhou L-Z, Wang J-G, Kombrink A, McCormick S, Zhang XS, et al Arabidopsis COBRA-LIKE 10, a GPI-anchored protein, mediates directional growth of pollen tubes. Plant J. 2013:74(3):486–497. 10.1111/tpj.12139 23384085

[kiad491-B67] Li YX , Qi BX. Progress toward understanding protein S-acylation: prospective in plants. Front Plant Sci. 2017:8:346. 10.3389/fpls.2017.00346 28392791 PMC5364179

[kiad491-B70] Lin DTS , Conibear E. ABHD17 Proteins are novel protein depalmitoylases that regulate N-Ras palmitate turnover and subcellular localization. Elife. 2015:4:e11306. 10.7554/eLife.11306 26701913 PMC4755737

[kiad491-B71] Lin S , Miao YJ, Huang HT, Zhang YT, Huang L, Cao JS. Arabinogalactan proteins: focus on the role in cellulose synthesis and deposition during plant cell wall biogenesis. Int J Mol Sci. 2022a:23(12):6578. 10.3390/ijms23126578 35743022 PMC9223364

[kiad491-B72] Lin W , Tang W, Pan X, Huang A, Gao X, Anderson CT, Yang Z. Arabidopsis pavement cell morphogenesis requires FERONIA binding to pectin for activation of ROP GTPase signaling. Curr Biol. 2022b:32(3):497–507.e4. 10.1016/j.cub.2021.11.030 34875229

[kiad491-B73] Lin Z , Xie F, Trivino M, Zhao T, Coppens F, Sterck L, Bosch M, Franklin-Tong VE, Nowack MK. Self-incompatibility requires GPI anchor remodeling by the poppy PGAP1 ortholog HLD1. Curr Biol. 2022c:32(9):1909–1923.e5. 10.1016/j.cub.2022.02.072 35316654 PMC7612714

[kiad491-B76] Liu E , MacMillan CP, Shafee T, Ma YX, Ratcliffe J, van de Meene A, Bacic A, Humphries J, Johnson KL. Fasciclin-like arabinogalactan-protein 16 (FLA16) is required for stem development in Arabidopsis. Front Plant Sci. 2020:11:615392. 10.3389/fpls.2020.615392 33362841 PMC7758453

[kiad491-B75] Liu J , Ishitani M, Halfter U, Kim CS, Zhu JK. The Arabidopsis thaliana SOS2 gene encodes a protein kinase that is required for salt tolerance. Proc Natl Acad Sci U S A. 2000:97(7):3730–3734. 10.1073/pnas.97.7.3730 10725382 PMC16308

[kiad491-B79] Liu L , Shang-Guan K, Zhang B, Liu X, Yan M, Zhang L, Shi Y, Zhang M, Qian Q, Li J, et al Brittle Culm1, a COBRA-like protein, functions in cellulose assembly through binding cellulose microfibrils. PLoS Genet. 2013:9(8):e1003704. 10.1371/journal.pgen.1003704 23990797 PMC3749933

[kiad491-B74] Liu X , Castro C, Wang YB, Noble J, Ponvert N, Bundy M, Hoel C, Shpak E, Palanivelu R. The role of LORELEI in pollen tube reception at the interface of the synergid cell and pollen tube requires the modified eight-cysteine motif and the receptor-like kinase FERONIA. Plant Cell. 2016a:28(5):1035–1052. 10.1105/tpc.15.00703 27081182 PMC4904665

[kiad491-B77] Liu X , Min L, Yang L, Chen ZA, Chun ZG, Ouyang YW, Zhao YW, Lin YX, Qi X, Yang CW, et al An ABHD17-like hydrolase screening system to identify de-S-acylation enzymes of protein substrates in plant cells. Plant Cell. 2021:33(10):3235–3249. 10.1093/plcell/koab199 34338800 PMC8505870

[kiad491-B78] Liu Z , Schneider R, Kesten C, Zhang Y, Somssich M, Zhang Y, Fernie AR, Persson S. Cellulose-microtubule uncoupling proteins prevent lateral displacement of microtubules during cellulose synthesis in Arabidopsis. Dev Cell. 2016b:38(3):305–315. 10.1016/j.devcel.2016.06.032 27477947

[kiad491-B80] Ma Y , MacMillan CP, de Vries L, Mansfield SD, Hao P, Ratcliffe J, Bacic A, Johnson KL. FLA11 and FLA12 glycoproteins fine-tune stem secondary wall properties in response to mechanical stresses. New Phytol. 2022:233(4):1750–1767. 10.1111/nph.17898 34862967 PMC9302641

[kiad491-B81] MacMillan CP , Mansfield SD, Stachurski ZH, Evans R, Southerton SG. Fasciclin-like arabinogalactan proteins: specialization for stem biomechanics and cell wall architecture in Arabidopsis and eucalyptus. Plant J. 2010:62(4):689–703. 10.1111/j.1365-313X.2010.04181.x 20202165

[kiad491-B82] Madson M , Dunand C, Li XM, Verma R, Vanzin GF, Calplan J, Shoue DA, Carpita NC, Reiter WD. The MUR3 gene of Arabidopsis encodes a xyloglucan galactosyltransferase that is evolutionarily related to animal exostosins. Plant Cell. 2003:15(7):1662–1670. 10.1105/tpc.009837 12837954 PMC165408

[kiad491-B83] Mahajan S , Pandey GK, Tuteja N. Calcium- and salt-stress signaling in plants: shedding light on SOS pathway. Arch Biochem Biophys. 2008:471(2):146–158. 10.1016/j.abb.2008.01.010 18241665

[kiad491-B84] Majeran W , Le Caer J-P, Ponnala L, Meinnel T, Giglione C. Targeted profiling of Arabidopsis thaliana subproteomes illuminates co- and posttranslationally N-terminal myristoylated proteins. Plant Cell. 2018:30(3):543–562. 10.1105/tpc.17.00523 29453228 PMC5894833

[kiad491-B85] MartiniÈre A , Moreau P. Complex roles of Rabs and SNAREs in the secretory pathway and plant development: a never-ending story. J Microsc. 2020:280(2):140–157. 10.1111/jmi.12952 32761815

[kiad491-B86] Matheson LA , Suri SS, Hanton SL, Chatre L, Brandizzi F. Correct targeting of plant ARF GTPases relies on distinct protein domains. Traffic. 2008:9(1):103–120. 10.1111/j.1600-0854.2007.00671.x 17988226

[kiad491-B87] McFarlane HE , Mutwil-Anderwald D, Verbancic J, Picard KL, Gookin TE, Froehlich A, Chakravorty D, Trindade LM, Alonso JM, Assmann SM, et al A G protein-coupled receptor-like module regulates cellulose synthase secretion from the endomembrane system in Arabidopsis. Dev Cell. 2021:56(10):1484–1497.e7. 10.1016/j.devcel.2021.03.031 33878345 PMC8754689

[kiad491-B88] Meents MJ , Motani S, Mansfield SD, Samuels AL. Organization of xylan production in the Golgi during secondary cell wall biosynthesis. Plant Physiol. 2019:181(2):527–546. 10.1104/pp.19.00715 31431513 PMC6776863

[kiad491-B89] Mortimer JC , Miles GP, Brown DM, Zhang ZN, Segura MP, Weimar T, Yu XL, Seffen KA, Stephens E, Turner SR, et al Absence of branches from xylan in Arabidopsis gux mutants reveals potential for simplification of lignocellulosic biomass. Proc Natl Acad Sci U S A. 2010:107(40):17409–17414. 10.1073/pnas.1005456107 20852069 PMC2951434

[kiad491-B90] Nakagawa Y , Katagiri T, Shinozaki K, Qi Z, Tatsumi H, Furuichi T, Kishigami A, Sokabe M, Kojima I, Sato S, et al Arabidopsis plasma membrane protein crucial for Ca2+ influx and touch sensing in roots. Proc Natl Acad Sci U S A. 2007:104(9):3639–3644. 10.1073/pnas.0607703104 17360695 PMC1802001

[kiad491-B91] Narváez-Barragán DA , Tovar-Herrera OE, Guevara-Garcia A, Serrano M, Martinez-Anaya C. Mechanisms of plant cell wall surveillance in response to pathogens, cell wall-derived ligands and the effect of expansins to infection resistance or susceptibility. Front Plant Sci. 2022:13:969343. 10.3389/fpls.2022.969343 36082287 PMC9445675

[kiad491-B92] Oxley D , Bacic A. Structure of the glycosylphosphatidylinositol anchor of an arabinogalactan protein from Pyrus communis suspension-cultured cells. Proc Natl Acad Sci U S A. 1999:96(25):14246–14251. 10.1073/pnas.96.25.14246 10588691 PMC24422

[kiad491-B93] Park HJ , Gamez-Arjona FM, Lindahl M, Aman R, Villalta I, Cha J-Y, Carranco R, Lim CJ, Garcia E, Bressan RA, et al S-acylated and nucleus-localized SALT OVERLY SENSITIVE3/CALCINEURIN B-LIKE4 stabilizes GIGANTEA to regulate Arabidopsis flowering time under salt stress. Plant Cell. 2023:35(1):298–317. 10.1093/plcell/koac289 36135824 PMC9806564

[kiad491-B94] Pierleoni A , Martelli PL, Casadio R. PredGPI: a GPI-anchor predictor. BMC Bioinformatics. 2008:9(1):392. 10.1186/1471-2105-9-392 18811934 PMC2571997

[kiad491-B95] Pierre M , Traverso JA, Boisson B, Domenichini S, Bouchez D, Giglione C, Meinnel T. N-myristoylation regulates the SnRK1 pathway in Arabidopsis. Plant Cell. 2007:19(9):2804–2821. 10.1105/tpc.107.051870 17827350 PMC2048702

[kiad491-B96] Polko JK , Barnes WJ, Voiniciuc C, Doctor S, Steinwand B, Hill JL, Tien M, Pauly M, Anderson CT, Kieber JJ. SHOU4 proteins regulate trafficking of cellulose synthase complexes to the plasma membrane. Curr Biol. 2018:28(19):3174–3182.e6. 10.1016/j.cub.2018.07.076 30245104

[kiad491-B97] Polko JK , Kieber JJ. The regulation of cellulose biosynthesis in plants. Plant Cell. 2019:31(2):282–296. 10.1105/tpc.18.00760 30647077 PMC6447023

[kiad491-B98] Preuss ML , Schmitz AJ, Thole JM, Bonner HKS, Otegui MS, Nielsen E. A role for the RabA4b effector protein PI-4 K beta 1 in polarized expansion of root hair cells in Arabidopsis thaliana. J Cell Biol. 2006:172(7):991–998. 10.1083/jcb.200508116 16567499 PMC2063757

[kiad491-B99] Renna L , Stefano G, Majeran W, Micalella C, Meinnel T, Giglione C, Brandizzi F. Golgi traffic and integrity depend on N-myristoyl transferase-1 in Arabidopsis. Plant Cell. 2013:25(5):1756–1773. 10.1105/tpc.113.111393 23673980 PMC3694704

[kiad491-B100] Rojek J , Tucker MR, Pinto SC, Rychlowski M, Lichocka M, Soukupova H, Nowakowska J, Bohdanowicz J, Surmacz G, Gutkowska M. Rab-dependent vesicular traffic affects female gametophyte development in Arabidopsis. J Exp Bot. 2021:72(2):320–340. 10.1093/jxb/eraa430 32939545 PMC7853608

[kiad491-B101] Roudier F , Schindelman G, DeSalle R, Benfey PN. The COBRA family of putative GPI-anchored proteins in Arabidopsis. A new fellowship in expansion. Plant Physiol. 2002:130(2):538–548. 10.1104/pp.007468 12376623 PMC166585

[kiad491-B102] Rui Y , Dinneny JR. A wall with integrity: surveillance and maintenance of the plant cell wall under stress. New Phytol. 2020:225(4):1428–1439. 10.1111/nph.16166 31486535

[kiad491-B103] Sato-Izawa K , Nakamura S-I, Matsumoto T. Mutation of rice bc1 gene affects internode elongation and induces delayed cell wall deposition in developing internodes. Plant Signal Behav. 2020:15(5):1749786. 10.1080/15592324.2020.1749786 32299283 PMC7238885

[kiad491-B104] Schindelman G , Morikami A, Jung J, Baskin TI, Carpita NC, Derbyshire P, McCann MC, Benfey PN. COBRA encodes a putative GPI-anchored protein, which is polarly localized and necessary for oriented cell expansion in Arabidopsis. Genes Dev. 2001:15(9):1115–1127. 10.1101/gad.879101 11331607 PMC312689

[kiad491-B105] Sedbrook JC , Carroll KL, Hung KF, Masson PH, Somerville CR. The Arabidopsis SKU5 gene encodes an extracellular glycosyl phosphatidylinositol-anchored glycoprotein involved in directional root growth. Plant Cell. 2002:14(7):1635–1648. 10.1105/tpc.002360 12119380 PMC150712

[kiad491-B106] Seifert GJ . Fascinating fasciclins: a surprisingly widespread family of proteins that mediate interactions between the cell exterior and the cell surface. Int J Mol Sci. 2018:19(6):1628. 10.3390/ijms19061628 29857505 PMC6032426

[kiad491-B107] Seifert GJ , Xue H, Acet T. The Arabidopsis thaliana fasciclin like arabinogalactan protein 4 gene acts synergistically with abscisic acid signalling to control root growth. Ann Bot. 2014:114(6):1125–1133. 10.1093/aob/mcu010 24603604 PMC4195540

[kiad491-B108] Shi HZ , Kim Y, Guo Y, Stevenson B, Zhu JK. The Arabidopsis SOS5 locus encodes a putative cell surface adhesion protein and is required for normal cell expansion. Plant Cell. 2003:15(1):19–32. 10.1105/tpc.007872 12509519 PMC143448

[kiad491-B109] Shi W , Zeng Q, Kunkel BN, Running MP. Arabidopsis Rab geranylgeranyltransferases demonstrate redundancy and broad substrate specificity in vitro. J Biol Chem. 2016:291(3):1398–1410. 10.1074/jbc.M115.673491 26589801 PMC4714223

[kiad491-B110] Shin Y , Chane A, Jung M, Lee Y. Recent advances in understanding the roles of pectin as an active participant in plant signaling networks. Plants (Basel). 2021:10(8):1712. 10.3390/plants10081712 34451757 PMC8399534

[kiad491-B111] Showalter AM , Keppler BD, Liu X, Lichtenberg J, Welch LR. Bioinformatic identification and analysis of hydroxyproline-rich glycoproteins in Populus trichocarpa. BMC Plant Biol. 2016:16(1):229. 10.1186/s12870-016-0912-3 27769192 PMC5073881

[kiad491-B112] Silva J , Ferraz R, Dupree P, Showalter AM, Coimbra S. Three decades of advances in arabinogalactan-protein biosynthesis. Front Plant Sci. 2020:11:610377. 10.3389/fpls.2020.610377 33384708 PMC7769824

[kiad491-B113] Srivastava V , Weber JR, Malm E, Fouke BW, Bulone V. Proteomic analysis of a poplar cell suspension culture suggests a major role of protein S-acylation in diverse cellular processes. Front Plant Sci. 2016:7:477. 10.3389/fpls.2016.00477 27148305 PMC4828459

[kiad491-B114] Stroppa N , Onelli E, Moreau P, Maneta-Peyret L, Berno V, Cammarota E, Ambrosini R, Caccianiga M, Scali M, Moscatelli A. Sterols and sphingolipids as new players in cell wall building and apical growth of Nicotiana tabacum L. pollen tubes. Plants (Basel). 2023:12(1):8. 10.3390/plants12010008 PMC9824051 36616135

[kiad491-B115] Sugiyama Y , Nagashima Y, Wakazaki M, Sato M, Toyooka K, Fukuda H, Oda Y. A Rho-actin signaling pathway shapes cell wall boundaries in Arabidopsis xylem vessels. Nat Commun. 2019:10(1):468. 10.1038/s41467-019-08396-7 30692538 PMC6349933

[kiad491-B116] Sun L , van Nocker S. Analysis of promoter activity of members of the PECTATE LYASE-LIKE (PLL) gene family in cell separation in Arabidopsis. BMC Plant Biol. 2010:10:152. 10.1186/1471-2229-10-152 20649977 PMC3017822

[kiad491-B117] Szumlanski AL , Nielsen E. The Rab GTPase RabA4d regulates pollen tube tip growth in Arabidopsis thaliana. Plant Cell. 2009:21(2):526–544. 10.1105/tpc.108.060277 19208902 PMC2660625

[kiad491-B118] Tan L , Eberhard S, Pattathil S, Warder C, Glushka J, Yuan CH, Hao ZY, Zhu X, Avci U, Miller JS, et al An Arabidopsis cell wall proteoglycan consists of pectin and arabinoxylan covalently linked to an arabinogalactan protein. Plant Cell. 2013:25(1):270–287. 10.1105/tpc.112.107334 23371948 PMC3584541

[kiad491-B119] Thinon E , Serwa RA, Broncel M, Brannigan JA, Brassat U, Wright MH, Heal WP, Wilkinson AJ, Mann DJ, Tate EW. Global profiling of co- and post-translationally N-myristoylated proteomes in human cells. Nat Commun. 2014:5:4919. 10.1038/ncomms5919 25255805 PMC4200515

[kiad491-B120] Thole JM , Perroud P-F, Quatrano RS, Running MP. Prenylation is required for polar cell elongation, cell adhesion, and differentiation in Physcomitrella patens. Plant J. 2014:78(3):441–451. 10.1111/tpj.12484 24634995

[kiad491-B121] Turupcu A , Almohamed W, Oostenbrink C, Seifert GJ. A speculation on the tandem fasciclin 1 repeat of FLA4 proteins in angiosperms. Plant Signal Behav. 2018:13(9):e1507403. 10.1080/15592324.2018.1507403 30148420 PMC6204788

[kiad491-B122] Vaddepalli P , Fulton L, Wieland J, Wassmer K, Schaeffer M, Ranf S, Schneitz K. The cell wall-localized atypical beta-1,3 glucanase ZERZAUST controls tissue morphogenesis in Arabidopsis thaliana. Development. 2017:144(12):2259–2269. 10.1242/dev.152231 28507000

[kiad491-B123] Vain T , Crowell EF, Timpano H, Biot E, Desprez T, Mansoori N, Trindade LM, Pagant S, Robert S, Hofte H, et al The cellulase KORRIGAN is part of the cellulose synthase complex. Plant Physiol. 2014:165(4):1521–1532. 10.1104/pp.114.241216 24948829 PMC4119035

[kiad491-B124] van de Meene AML , Doblin MS, Bacic A. The plant secretory pathway seen through the lens of the cell wall. Protoplasma. 2017:254(1):75–94. 10.1007/s00709-016-0952-4 26993347

[kiad491-B125] Vellosillo T , Dinneny JR, Somerville CR, Ehrhardt DW. TRANVIA (TVA) facilitates cellulose synthase trafficking and delivery to the plasma membrane. Proc Natl Acad Sci U S A. 2021:118(30):e2021790118. 10.1073/pnas.2021790118 34290139 PMC8325160

[kiad491-B126] Vogel JP , Raab TK, Schiff C, Somerville SC. PMR6, A pectate lyase-like gene required for powdery mildew susceptibility in Arabidopsis. Plant Cell. 2002:14(9):2095–2106. 10.1105/tpc.003509 12215508 PMC150758

[kiad491-B127] Voiniciuc C , Schmidt MH-W, Berger A, Yang B, Ebert B, Scheller HV, North HM, Usadel B, Guenl M. MUCILAGE-RELATED10 produces galactoglucomannan that maintains pectin and cellulose architecture in Arabidopsis seed mucilage. Plant Physiol. 2015:169(1):403–420. 10.1104/pp.15.00851 26220953 PMC4577422

[kiad491-B128] Voragen AGJ , Coenen G-J, Verhoef RP, Schols HA. Pectin, a versatile polysaccharide present in plant cell walls. Struct Chem. 2009:20(2):263–275. 10.1007/s11224-009-9442-z

[kiad491-B132] Wang DD , Yeats TH, Uluisik S, Rose JKC, Seymour GB. Fruit softening: revisiting the role of pectin. Trends Plant Sci. 2018:23(4):302–310. 10.1016/j.tplants.2018.01.006 29429585

[kiad491-B129] Wang T , McFarlane HE, Persson S. The impact of abiotic factors on cellulose synthesis. J Exp Bot. 2016:67(2):543–552. 10.1093/jxb/erv488 26552883

[kiad491-B131] Wang Y , Yang W. Proteome-scale analysis of protein S-acylation comes of age. J Proteome Res. 2021:20(1):14–26. 10.1021/acs.jproteome.0c00409 33253586 PMC7775881

[kiad491-B130] Wang YY , Su ZX, Gu X. What is the main mechanism of the origin of phosphorylation sites? Still an open question. J Syst Evol. 2017:55(3):231–234. 10.1111/jse.12244

[kiad491-B133] Wu H-C , Bulgakov VP, Jinn T-L. Pectin methylesterases: cell wall remodeling proteins are required for plant response to heat stress. Front Plant Sci. 2018:9:1612. 10.3389/fpls.2018.01612 30459794 PMC6232315

[kiad491-B135] Xu H , Giannetti A, Sugiyama Y, Zheng W, Schneider R, Watanabe Y, Oda Y, Persson S. Secondary cell wall patterning-connecting the dots, pits and helices. Open Biol. 2022a:12(5):210208. 10.1098/rsob.210208 35506204 PMC9065968

[kiad491-B134] Xu Z , Gao YH, Gao CX, Mei JS, Wang SG, Ma JX, Yang HL, Cao SX, Wang Y, Zhang FX, et al Glycosylphosphatidylinositol anchor lipid remodeling directs proteins to the plasma membrane and governs cell wall mechanics. Plant Cell. 2022b:34(12):4778–4794. 10.1093/plcell/koac257 35976113 PMC9709986

[kiad491-B136] Xue H , Veit C, Abas L, Tryfona T, Maresch D, Ricardi MM, Estevez JM, Strasser R, Seifert GJ. Arabidopsis thaliana FLA4 functions as a glycan-stabilized soluble factor via its carboxy-proximal fasciclin 1 domain. Plant J. 2017:91(4):613–630. 10.1111/tpj.13591 28482115 PMC5575511

[kiad491-B137] Yeats TH , Somerville CR. A dual mechanism of cellulose deficiency in *shv3svl1*. Plant Signal Behav. 2016:11:3. 10.1080/15592324.2016.1218108 PMC5155455 27494413

[kiad491-B138] Zhang X , Bian A, Li T, Ren L, Li L, Su Y, Zhang Q. ROS And calcium oscillations are required for polarized root hair growth. Plant Signal Behav. 2022:17(1):2106410. 10.1080/15592324.2022.2106410 35938584 PMC9359386

[kiad491-B139] Zhang YL , Li E, Feng QN, Zhao XY, Ge FR, Zhang Y, Li S. Protein palmitoylation is critical for the polar growth of root hairs in Arabidopsis. BMC Plant Biol. 2015:15:50. 10.1186/s12870-015-0441-5 25849075 PMC4340681

[kiad491-B140] Zheng L , Liu P, Liu Q, Wang T, Dong J. Dynamic protein S-acylation in plants. Int J Mol Sci. 2019:20(3):560. 10.3390/ijms20030560 30699892 PMC6387154

[kiad491-B141] Zhong RQ , Cui DT, Ye ZH. Secondary cell wall biosynthesis. New Phytol. 2019:221(4):1703–1723. 10.1111/nph.15537 30312479

[kiad491-B142] Zhou K . GPI-anchored SKS proteins regulate root development through controlling cell polar expansion and cell wall synthesis. Biochem Biophys Res Commun. 2019:509(1):119–124. 10.1016/j.bbrc.2018.12.081 30578078

[kiad491-B143] Zhou K . The regulation of the cell wall by glycosylphosphatidylinositol-anchored proteins in Arabidopsis. Front Cell Dev Biol. 2022:10:904714. 10.3389/fcell.2022.904714 36036018 PMC9412048

[kiad491-B144] Zhou YL , Yang Y, Niu Y, Fan TT, Qian D, Luo CX, Shi YM, Li SW, An LZ, Xiang Y. The tip-localized phosphatidylserine established by Arabidopsis ALA3 is crucial for Rab GTPase-mediated vesicle trafficking and pollen tube growth. Plant Cell. 2020:32(10):3170–3187. 10.1105/tpc.19.00844 32817253 PMC7534478

[kiad491-B145] Zhu Y , McFarlane HE. Regulation of cellulose synthesis via exocytosis and endocytosis. Curr Opin Plant Biol. 2022:69:102273. 10.1016/j.pbi.2022.102273 35987011

